# Synergistic Interplay between Curcumin and Polyphenol-Rich Foods in the Mediterranean Diet: Therapeutic Prospects for Neurofibromatosis 1 Patients

**DOI:** 10.3390/nu9070783

**Published:** 2017-07-21

**Authors:** Teresa Esposito, Carla Schettino, Paola Polverino, Salvatore Allocca, Laura Adelfi, Alessandra D’Amico, Guglielmo Capaldo, Bruno Varriale, Anna Di Salle, Gianfranco Peluso, Giuseppe Sorrentino, Giacomo Lus, Simone Sampaolo, Giuseppe Di Iorio, Mariarosa Anna Beatrice Melone

**Affiliations:** 1 Department of Experimental Medicine, Section of Human Physiology and Unit of Dietetics and Sports Medicine, University of Campania Luigi Vanvitelli, via Santa Maria di Costantinopoli 16, 80138 Naples, Italy; teresa.esposito@unicampania.it (T.E.); alloccasalvatore2016@libero.it (S.A.); laura.adelfi@hotmail.it (L.A.); 2 Division of Neurology, Center for Rare Diseases & Inter University Center for Research in Neurosciences, Department of Medical, Surgical, Neurologic, Metabolic and Aging Sciences, University of Campania Luigi Vanvitelli, via Sergio Pansini, 5, 80131 Naples, Italy; carla.schettino@unicampania.it (C.S.); polverinopaola@gmail.com (P.P.); dr.guglielmo.capaldo@virgilio.it (G.C.); giacomo.lus@unicampania.it (G.L.); simone.sampaolo@unicampania.it (S.S.); giuseppe.diiorio@unicampania.it (G.D.I.); 3 Department of Advanced Biomedical Sciences, Neuroradiology, University “Federico II”, via Pansini, 5, 80131 Naples, Italy; damicoalex@tiscali.it; 4 Department of Experimental Medicine, Laboratory of Molecular Biology and Genetics, University of Campania Luigi Vanvitelli, via Santa Maria di Costantinopoli 16, 80138 Naples, Italy; bruno.varriale@unicampania.it; 5 Institute of Agro-environmental and Forest Biology, National Research Council, via Pietro Castellino 111, 80131 Naples, Italy; anna.disalle@cnr.it (A.D.S.); gianfranco.peluso@cnr.it (G.P.); 6 ISAFOM, National Research Council, via Patacca 85, Ercolano (NA), 80056 Naples, Italy; giuseppe.sorrentino@isafom.cnr.it

**Keywords:** neurofibromatosis 1, neurofibromas, curcumin, diet, polyphenols

## Abstract

Neurofibromas are the hallmark lesions in Neurofibromatosis 1 (NF1); these tumors are classified as cutaneous, subcutaneous and plexiform. In contrast to cutaneous and subcutaneous neurofibromas, plexiform neurofibromas can grow quickly and progress to malignancy. Curcumin, a turmeric-derived polyphenol, has been shown to interact with several molecular targets implicated in carcinogenesis. Here, we describe the impact of different dietary patterns, namely Mediterranean diet (MedDiet) compared to the Western diet (WesDiet), both with or without curcumin, on NF1 patients’ health. After six months, patients adopting a traditional MedDiet enriched with 1200 mg curcumin per day (MedDietCurcumin) presented a significant reduction in the number and volume of cutaneous neurofibromas; these results were confirmed in subsequent evaluations. Notably, in one patient, a large cranial plexiform neurofibroma exhibited a reduction in volume (28%) confirmed by Magnetic Resonance Imaging. Conversely, neither unenriched MedDiet nor WesDiet enriched with curcumin exhibited any significant positive effect. We hypothesize that the combination of a polyphenol-rich Mediterranean diet and curcumin was responsible for the beneficial effect observed on NF1. This is, to the best of our knowledge, the first experience with curcumin supplementation in NF1 patients. Our report suggests that an integrated nutritional approach may effectively aid in the management of NF1.

## 1. Introduction

Polyphenols are secondary metabolites ubiquitous in the plant kingdom with unique antioxidant properties and a wide spectrum of therapeutic activities. It is well known that the phenolic compounds present mainly in olive oil contribute significantly to the superior health profile observed in Mediterranean populations following the traditional Mediterranean diet (MedDiet). In addition, curcumin, the most bioactive polyphenolic constituent of turmeric and an integral part of the Indian diet, exerts protective effects on a variety of diseases such as cardiac diseases, cancer, diabetes, Alzheimer’s disease, rheumatoid arthritis and psoriasis [1,2,3,4,5,6].

However, the mechanisms by which dietary polyphenols can affect human diseases are multiple and complex. Various stages of carcinogenesis may be inhibited by polyphenols in in vitro or in vivo systems. For example, curcumin is able to inhibit diethylnitrosamine-induced hepatocarcinogenesis in mice at a concentration of 0.2% in the diet [7], but the biological mechanism(s) of this effect has only been partially clarified. Again, olive oil polyphenols seem to exert anticancer effects through the modulation of genes and molecular signaling pathways associated with cell survival, cell cycle progression, cell growth arrest and apoptosis, as demonstrated in several tumor cell lines [8]. Recent findings suggest that polyphenols may exert anti-proliferative activity by affecting the cell metabolism. In particular, polyphenols have the potential to modulate glucose uptake, and to alter the glutathione as well as lipid metabolism [9]. Furthermore, there is experimental evidence that supports an effect of polyphenols on the entry of glutamine, an amino-acid essential for cancer metabolism, into the cells [10].

Interestingly, it has also been reported that polyphenols can affect tumor progression by interfering with the dynamic interactions between several components within the tumor microenvironment and the cancer cells. Indeed, the bi-directional interaction between cancer cells and the surrounding cells increases tumor proliferation, invasion and metastasis, and allows the tumor cells to resist therapeutic insults [11]. Curcumin is able to interfere with the cross-talk between cancer stem cells and stromal fibroblasts, resulting in the reversal of epithelial to mesenchymal transition and associated metastasis [11]. Furthermore, curcumin, by a dual mode of action, is able to modulate the enzymatic activity of the EGF receptor (EGF-R) intracellular domain [12].

In our study, we evaluated the effects of a traditional Mediterranean diet (rich in polyphenol content) compared to a Western dietary “pattern”, both with or without curcumin supplementation, in case series of patients with Neurofibromatosis type 1 (NF1), one of the most common autosomal dominant genetic disorders that affects ~1 in 3500 individuals [13]. The NF1 gene (17q11.2.5; NM_000267.3) encodes neurofibromin, a cytoplasmic protein of 2818 amino acids (molecular mass of 220–250 kDa) that promotes the intrinsic GTPase activity of Ras [14]. Nearly all NF1 patients develop dermal neurofibromas. In addition, they can develop brain tumors (gliomas and glioblastomas) and peripheral nerve tumors (spinal neurofibromas, plexiform neurofibromas and malignant peripheral nerve sheath tumors). The NF1 phenotype is not complete at birth despite being a genetic disease [13,14]. The mutation of NF1 has full penetrance and the symptoms appear in an age-dependent manner [13,14]. Therefore, the influence of nutrition is fundamental in the epigenetic control of the disease. Of interest, de Souza et al, reporting that NF1 patients consumed an unhealthy diet, rich in fats and sodium and poor in fiber, vitamins, and minerals, hypothesized a role of the dietary and nutritional patterns in the severity of the clinical manifestations of NF1 [15]. In addition, Carotenuto and Esposito demonstrated that adjustments of the diet influenced the clinical features in NF1. In particular, the addition of nutraceuticals improved several symptoms affecting NF1 patients, such as migraine-related disability [16]. Although considerable progress has been made in understanding NF1, no effective therapy is available to treat this pathological condition. Herein we demonstrated that a Mediterranean diet and curcumin act synergically to induce a significant reduction in the number and size of neurofibromas, thus suggesting that an integrated nutritional approach could be effective in the management of NF1.

## 2. Materials and Methods

Our series included eleven NF1 patients identified on the basis of the National Institutes of Health (NIH) Consensus Conference criteria (National Institutes of Health Consensus Development Conference Statement: Neurofibromatosis 1988), clinically followed up at our Division of Neurology, Neurofibromatosis and Rare Diseases Center of the University Hospital, University of Campania Luigi Vanvitelli, Italy (Table 1). Of these eleven patients, seven lacked a family history of NF1.

Eight patients were screened for NF1 mutations (see Table 1). Genetic testing revealed that six of them carried a mutation producing truncated neurofibromin, as confirmed by a new diagnostic technique recently described by our group [17]; Patient 9 presented a single-nucleotide mutation, while Patient 3 presented a newly discovered intragenic heterozygous deletion encompassing exon 12 and 13 [18].

### 2.1. Methods

The NF1 patients were divided into four groups on the basis of their dietary regimen: the first group (*n* = 2) was instructed to follow a Western diet (WesDiet); the second (*n* = 3) a traditional Mediterranean-style diet (MedDiet); the third (*n* = 3) a Western diet enriched with curcumin (WesDietCurcumin); and the fourth (*n* = 3) a traditional Mediterranean-style diet enriched with curcumin (MedDietCurcumin). All the diets were evaluated by a dietitian. In particular, the Western diet included ad libitum the consumption of red and processed meat, refined grains, French fries, sweets and desserts. No total calorie restriction was advised.

The traditional Mediterranean-style diet included the abundant use of olive oil for cooking and dressing dishes (extra-virgin olive oil—50 mL/day with >2000 mg/kg total phenol concentration as determined by Folin-Ciocalteu’s assay—Sigma Aldrich, St. Louis, MO, USA), the consumption of ≥2 daily servings of vegetables, ≥3 daily servings of fresh fruits, ≥3 weekly servings of legumes, ≥3 weekly servings of fish or seafood, white meats instead of red meats or processed meats (burgers, sausages), and avoiding the consumption of cream, butter, margarine, pate, duck, carbonated and/or sugared beverages, pastries, industrial bakery products and desserts, French fries or potato chips, and sweets. Curcumin was supplemented at a dose of 1200 mg to the third and fourth groups at lunchtime [19,20]. Finally, all patients were invited to restrict foods known to contain curcumin.

Physical activity was not specifically advised for all NF1 patients. Compliance with the program was assessed by attendance at the follow-up visits and completion of the diet diaries. The patients were seen at two-monthly intervals over the six months of follow-up, after the introduction of the different diets. A complete physical examination, an evaluation of plasmatic curcumin level, integrated by photographic documentation were performed at each visit. Manual counting one by one of cutaneous neurofibromas was performed on all images (to facilitate the counting process, images were re-elaborated using Photoshop^®^ software by Adobe Systems Software, Belfast, Ireland). 

When appropriate, magnetic resonance imaging (MRI) was performed. Head MR was performed using a 1.5 T MRI unit (Philips Gyroscan) including axial Spin-Echo T1, Turbo Spin-Echo (TSE) T2 and FLAIR (slice thickness: 5 mm), coronal FLAIR (slice thickness 3 mm), sagittal TSE T2 (slice thickness: 4 mm) and axial DWI (Diffusion Weighted Imaging). Volumetric analysis was performed using an algorithm for volumetric evaluations available in OsiriX, a software for viewing and processing DICOM images. It was necessary to make a correction of the gap between the slices (intergap correction). The tumor profiles were manually traced “slice by slice”.

The patients’ nutritional status was evaluated applying a combination of clinical observation, bioimpedentiometric analysis and anthropometric and biochemical parameters.

### 2.2. Ethics

This study was approved by the Medical Ethics Committee and Safety Board of the University of Campania Luigi Vanvitelli, in accordance with the Declaration of Helsinki on ethical principles for medical research involving human subjects (protocol number 479/13). Written informed consent was obtained from each patient before admission to the study and to allow the initiation of any study-related procedures. In addition, the patients authorized the publication of this manuscript, its accompanying images and other data.

### 2.3. Plasma Sample Preparation

A plasma aliquot was first treated with 10 μL of 6.0 M HCl and then with 10 U of β-glucuronidase type H-1 from Helix pomatia in 0.1 M phosphate buffer (pH 6.86). The resulting mixture was then thoroughly vortexed and incubated at 37 °C for 1 h to hydrolyze the phase-2 conjugates of curcuminoids. After incubation, curcuminoids were extracted with 2 volumes of methanol/chloroform (1:2 *v*/*v*), sonicated in a water bath for 15 min and evaporated to dryness at 30 °C under negative pressure in a centrifugal concentrator. This process was repeated for a total of two extractions. The dried extract was reconstituted in methanol and subjected to HPLC analysis.

### 2.4. Chromatographic Analysis of Curcuminoids

The HPLC-UV procedure was conducted according to Heath et al. [21] with some modification. The analysis was carried out on Agilent 1260 Infinity Quaternary LC (Agilent Technologies, Santa Clara, CA, USA) equipped with a DAD (Diode-Array Detector). The chromatographic separation was performed on a Gemini^®^ 5 µm C18 110 Å, LC Column 250 × 4.6 mm (Phenomenex, Torrance, CA, USA) protected by a guard column (Security Guard Cartridge C18, 4 × 2.0 mm inner diameter, Phenomenex, Torrance, CA) and maintained at 30 °C. A linear elution gradient consisting of a mobile phase A (0.1% acetic acid), B (Acetonitrile), and C (Methanol) was programmed as follows: initially 50% A, 45% B, and 5% C, linearly changed to 30% A, 65% B, and 5% C over 5 min, and then held for 4 min at 30% A, 65% B, and 5% C. The system was then re-equilibrated for 5 min with the initial solvent. The detection wavelength was set at 420 nm. The quantitation of curcuminoids is by peak area ratio (curcumin, demethoxycurcumin and bisdemethoxycurcumin to internal standard) and is based on a standard curve in a plasma or urine matrix, generated by using an external standard to spike plasma or urine. A linear curve is generated from a single analysis of six different standard concentrations. System control and data acquisition were performed using the ChemStation software (Agilent Technologies, Santa Clara, CA, USA).

### 2.5. Statistical Analysis

Statistical analysis was conducted using a one-way ANOVA test, with the significant differences determined at *p* < 0.05, using GraphPad Prism Version 5.04 software (GraphPad Software, San Diego, CA, USA).

## 3. Results

Patients 1, 2, 6, 7 and 8 did not adhere to the MedDiet and did not significantly modify their dietary habits, which were consistent with a WesDiet; among them, Patient 6, 7 and 8 agreed to introduce curcumin to their diet (WesDietCurcumin). Patients 3, 4 and 5 followed a MedDiet. Patients 9, 10 and 11 agreed to follow a MedDietCurcumin. As shown in Table 1, all patients presented a clinical picture characterized by more than six cafè-au-lait spots, intertriginous freckling and both cutaneous and subcutaneous neurofibromas; patients 2, 3, 4, 7 and 11 presented plexiform neurofibromas. NF1 complications were present in patients 1, 2, 4, 5, 6, 7 and 11 with scoliosis, in patient 4 with short stature, and in patients 1 and 2 with optic pathway glioma [22]. Patients 3, 6 and 8 presented cerebrovascular abnormalities, which are a common occurrence in NF1 [23]. In particular, patient 3 presented very rare bilateral aneurysms of both internal carotid arteries, as extensively described in a previous report [18]. Patients 1, 2, 3 and 4 had a family history of NF1.

### 3.1. Follow-Up

**Figure 1:**
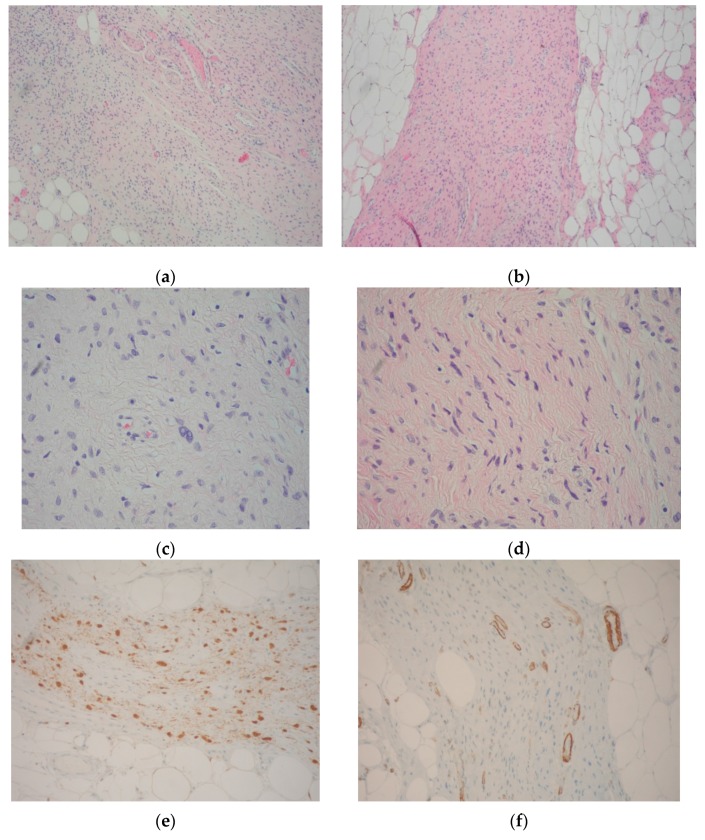

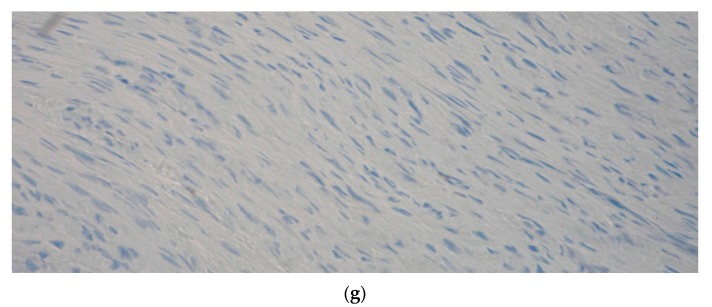
Neurofibroma resection specimen. (**a**,**b**) Spindle “wavy” cells in a matrix of fine fibrillary collagen; neurofibromatous tissue merges with mature fat and ectatic vessels (Hematoxylin-eosin, Magnification 10×); (**c**,**d**) Spindle cell nuclei in a fine fibrillary matrix, at a greater magnification (Hematoxylin-Eosin, Magnification 40×); (**e**) S100 immunohistochemical test confirmed the neurogenic origin of the lesion (Magnification 20×); (**f**) Actin-hhf35 immunohistochemical test excluded the myogenic origin of the lesion (note that only perivascular splindle smooth cells were reactive) (Magnification 20×); (**g**) Proliferation index by Ki67 immunohistochemical test was almost negative (Magnification 20×).

**Figure 2:**
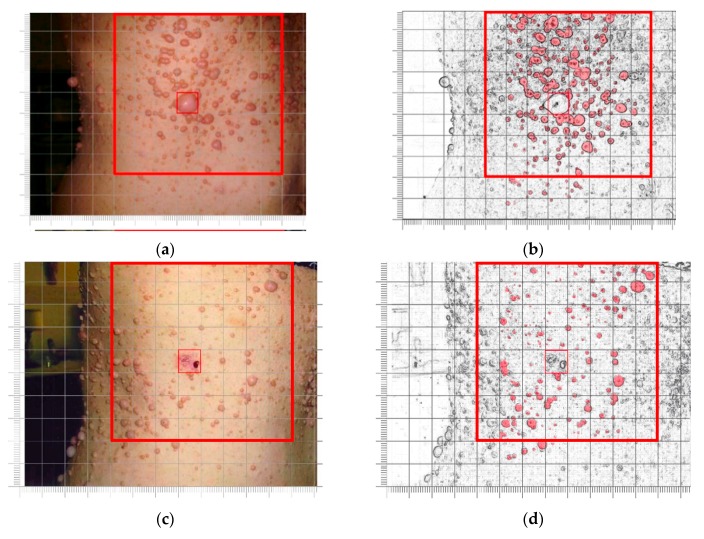

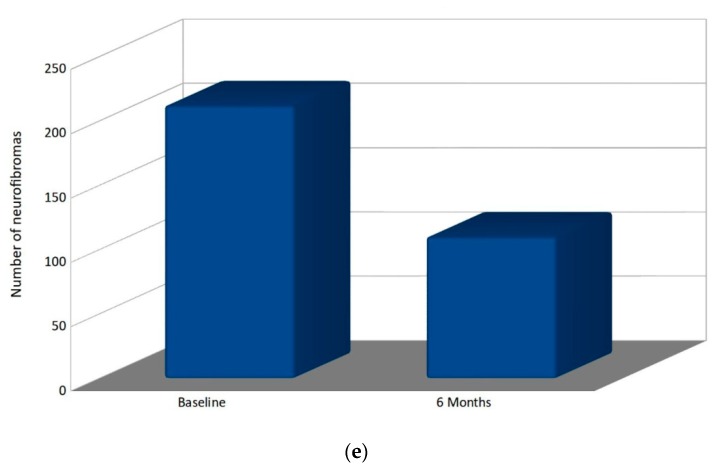
Right lateral view of thoracic region in patient 9: serial clinical assessment. (**a**,**c**) The large red square outlines the sampled area at baseline (**a**) and after six months of MedDietCurcumin (**c**); the small red square indicates the most prominent neurofibroma in the area, which was removed immediately after baseline observation for histological examination; (**b**,**d**) Digital re-elaboration of the images in A and C to facilitate manual counting of cutaneous neurofibromas; (**e**) Bar chart representing the number of neurofibromas in the sampled area at baseline and at six-month follow-up.

Clinical follow-up integrated by self-assessment of outcome in the first (WesDiet—patients 1 and 2), second (MedDiet—patients 3, 4 and 5) and third group (WesDietCurcumin—patients 6, 7 and 8) did not detect significant phenotypic variations in the number, size and color of cutaneous neurofibromas or in other signs and symptoms of disease, clinically detectable, at the different time points, compared to baseline. Patient 9 (Table 1), a 54-year-old woman, showed a severe cutaneous phenotype. Her medical history was notable for numerous surgical removals of limb and trunk lesions, histologically defined as neurofibromas (Figure 1a–g). A comprehensive physical examination performed at baseline showed widespread cafè-au-lait spots, axillary and inguinal freckling and numerous cutaneous neurofibromas on the neck, trunk, upper and lower limbs and perianal area. These neurofibromas varied in size and shape, with both sessile and pedunculated forms widely represented; they were soft and not painful to the touch. While most smaller neurofibromas were flesh-colored, the area located between the right anterior and posterior axillary lines comprised several tumors with a brownish-red color; a larger, round, pedunculated neurofibroma was particularly prominent (Figure 2a). In this area a manual count of 212 distinct neurofibromas was made (Figure 2b). Six months after the introduction of MedDietCurcumin, as shown in Figure 2c,d, there was a striking reduction in the number of neurofibromas compared to baseline. In fact, at this follow-up appointment, only 110 (51%) neurofibromas were detected (Figure 2f). Besides a decrease in the number of neurofibromas, a decrease in their volume was observed, particularly evident for the larger neurofibromas, which appeared to be considerably smaller in size, lighter in color, and softer to the touch.

**Figure 3:**
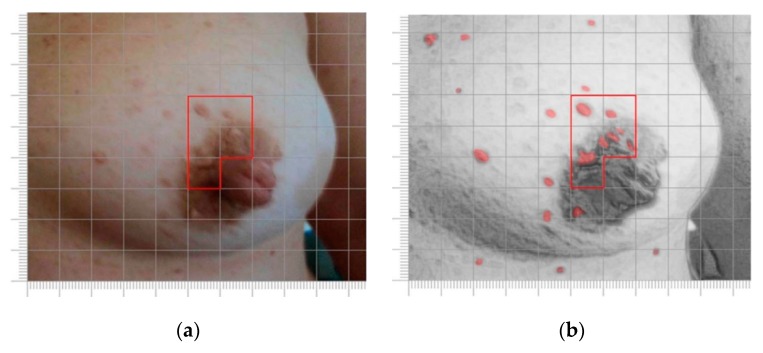

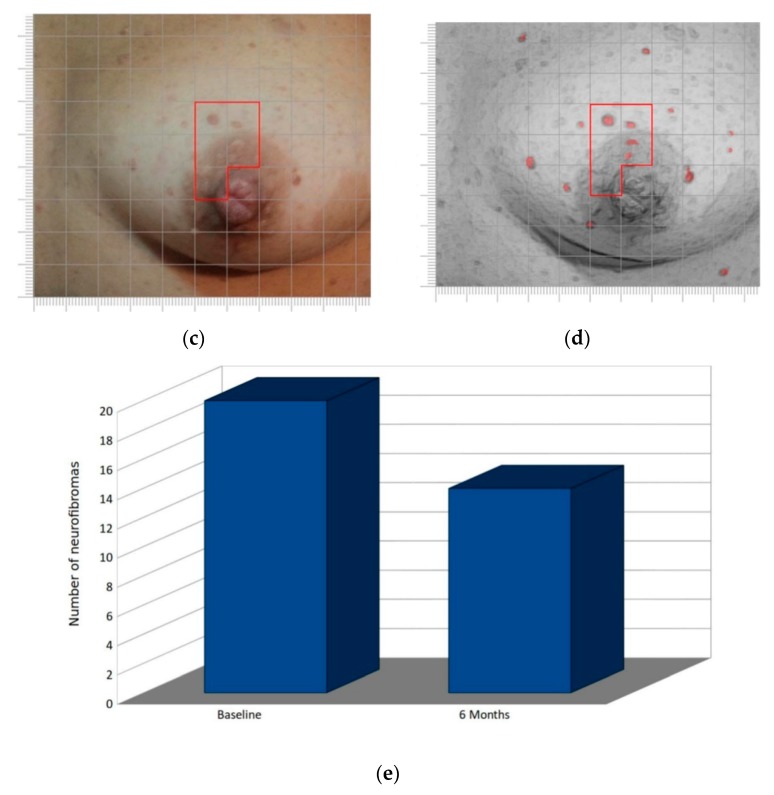
Left breast of case 10: serial clinical assessment. (**a**,**c**). Clinical observation at baseline (**a**) and after six months of MedDietCurcumin (**c**); the red border is provided for reference; (**b**,**d**) Digital reworking of the image in A and C to facilitate manual counting of cutaneous neurofibromas; (**e**) Bar chart representing the number of neurofibromas in the sampled area at baseline and at six-month follow-up.

Patient 10, (Table 1), a 27-year-old woman diagnosed in infancy due to the presence of more than six cafè-au-lait spots; during adolescence, she developed axillary freckling and several neurofibromas. Prior to the introduction of MedDietCurcumin, she presented diffuse cafè-au-lait spots, bilateral axillary and inguinal freckling and cutaneous neurofibromas of various sizes on the trunk and limbs. Several sessile and pedunculated cutaneous neurofibromas were observed on the left breast, and in particular in the areolar area (Figure 3a); in the area selected, we counted 20 neurofibromas (Figure 3b). At the six-month follow-up, she presented a reduction in the volume of several cutaneous neurofibromas in the area selected. Furthermore, a significant decrease in the number of neurofibromas (30%) was evident, as shown in Figure 3c,d and in the histogram in Figure 3e.

**Figure 4:**
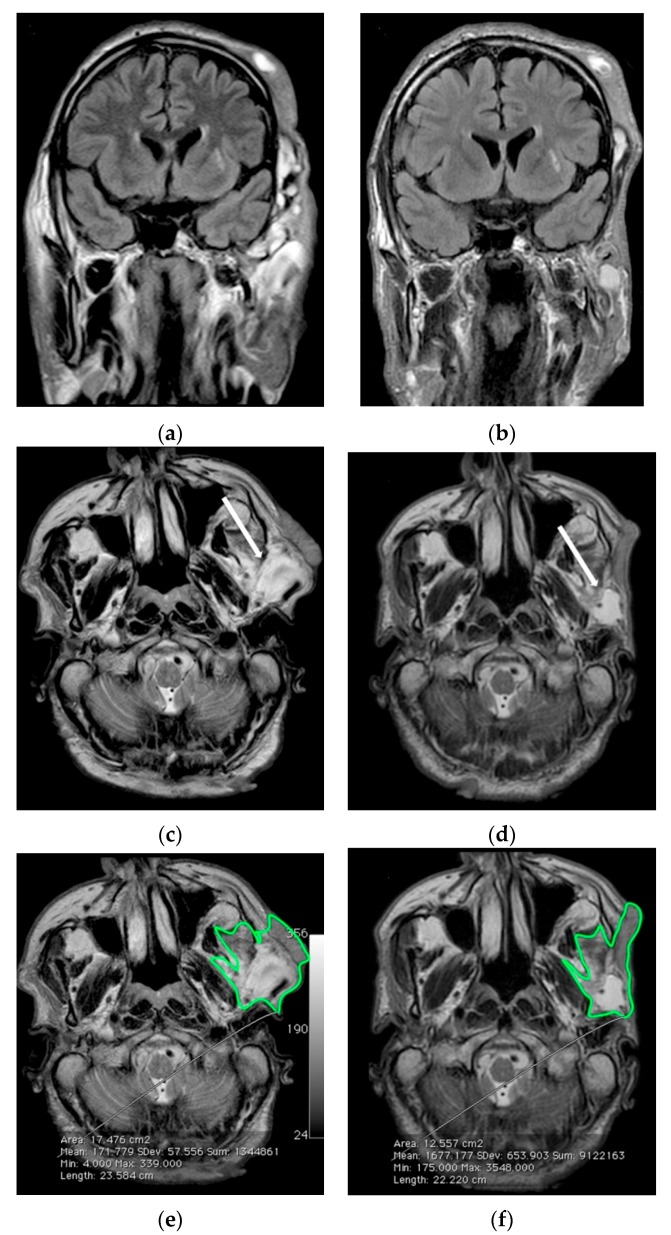
Serial MR imaging assessment of case 11. (**a**) Coronal FLAIR-weighted and (**c**,**e**) Axial TSE T2-weighted images of the left facial plexiform neurofibroma at baseline; (**b**) Coronal FLAIR-weighted and (**d**,**f**) Axial TSE T2-weighted images after six months of MedDietCurcumin. After six months there was a significant reduction especially in the hyperintense parts of the lesion, as indicated by arrows in (**c**,**d**). In (**e**,**f**) a green border contours the area used for volume measurement.

Patient 11 (Table 1), a 50-year-old man at the time of our first observation. Since childhood, he presented cafè-au-lait spots and subcutaneous neurofibromas on the trunk and lower limbs; he had also developed a large formation in the left orbito-temporal region, which had been partially resected when he was 44 years old; this lesion had been histologically diagnosed as a plexiform neurofibroma. During the following two years, the residual neurofibroma had increased in volume, infiltrating the ipsilateral orbital cavity and compressing the eyeball and causing partial left palpebral ptosis. Clinical examination of the lesion at baseline showed a large subcutaneous mass originating from the left upper eyelid and reaching the temporal region. It had a gray-rosy color, indistinct margins, soft elastic consistency and was not painful to the touch. A brain MRI at baseline (Figure 4a,c,e) showed pseudo-nodular formations (neurofibromas) in the left fronto-parietal-temporal area, extending to the ipsilateral orbital region. Six months later, the brain MRI (Figure 4b,d,f) showed a clear volume reduction in this plexiform neurofibroma, especially visible in axial TSE T2 weighted slices of the inferior portion (Figure 4e,f). The total volume reduction was around 28%.

### 3.2. Nutritional and Bioimpedentiometric Data

From a metabolic point of view, as shown in Table 2, no significant changes in BMI in the WesDiet group (patients 1 and 2) or WesDietCurcumin (6–8) were observed.

All patients following a Mediterranean diet with curcumin supplementation (9, 10 and 11) or without curcumin (3, 4 and 5) presented a general improvement in their metabolic status, with a BMI reduction and an increase in body hydration, due to the greater intake of water; in all of these patients, the percentage of fat-free mass (FFM), and basal metabolism (BMR) increased. Phase angle (PA) improved in the Mediterranean and MedDietCurcumin patients, with the exception of patient 11, for whom it remained stable. Na/K exchange remained essentially unchanged in all of these patients.

No patient reported any side effects of curcumin consumption

### 3.3. Plasma Curcuminoid Level

**Figure 5:**
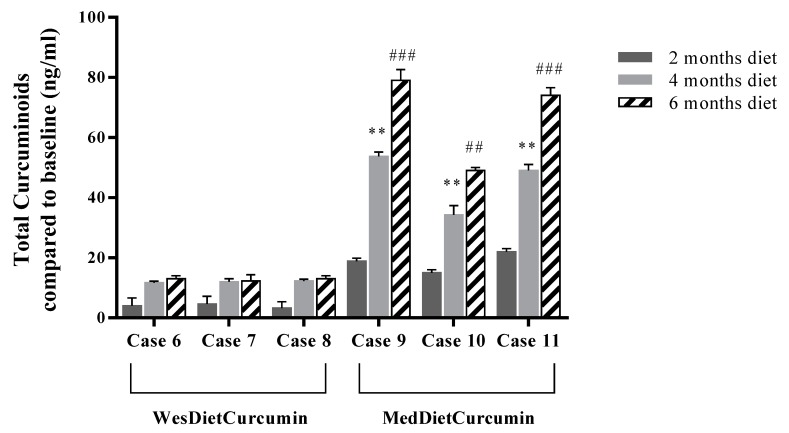
Plasma levels of curcuminoids. HPLC analysis of enzymatically hydrolyzed plasma samples. For each sample, three different experiments were conducted and the results expressed as the mean of the values obtained (mean ± SD). Statistically significant variations: ** *p* < 0.01 4 months diet versus 2 months diet; ## *p* < 0.01 6 months diet versus 2 months diet; ### *p* < 0.001 6 months diet versus 2 months diet.

The curcumin supplemented dietary regimen was established on the basis of previous clinical studies for inflammatory conditions, where active dosages of around 1–2 g/day of curcuminoid were used [19,20]. For this study, subjects consumed, in association with Western or traditional Mediterranean-style diet, 1200 mg total curcuminoids. Once absorbed, curcumin is subjected to conjugations like sulfation and glucuronidation at various tissue sites. For this reason, all plasma samples were treated with Helix pomatia glucuronidase/sulfatase before HPLC analysis. Our data (Figure 5) demonstrated that the plasma level of curcumin increased linearly over sixth months in patients who consumed the MedDietCurcumin, reaching a final plasma concentration ranging from 49.2 ± 1.0 ng/mL to 79.0 ± 3.6 ng/mL. Conversely, patients treated with a Western diet supplemented with curcumin showed very low plasma concentrations of curcumin even after sixth months of diet. Taken together, our results demonstrate that a traditional Mediterranean-style diet improves curcuminoid bioavailability.

## 4. Discussion

The association between NF1 and malignant tumors (gliomas, malignant peripheral nerve sheath tumors (MPNST), leukemia and rhabdomyosarcoma) has been largely described [24]. The mutation is highly penetrant: by age 20, almost 100% of the mutation carriers will manifest the disease in some form [25]. NF1 is notable for its high phenotypic variability, both within and between families, which means that family members carrying the exact same mutation may present with vastly different clinical pictures [26]. Multiple factors have been put forward as mechanisms underlying this variability, including modifying genes [27], allelic heterogeneity, mutation in the second allele, somatic mosaicism, epigenetic events and exposure to environmental agents [28,29]. 

Here we report the beneficial effects of a traditional Mediterranean Diet (MedDiet) enriched with curcumin (MedDietCurcumin) on the number and size of neurofibromas in all NF1 patients consuming this diet (patients 9–11). Indeed, besides the improvement in the general metabolic status, and particularly of the lipid profile, we observed a marked reduction in the number (ranging from 30 to 51%) and volume of neurofibromas after six months of MedDietCurcumin. Notably, the large cranial plexiform neurofibroma in patient 11 exhibited a marked volume reduction (28.2%), as shown by a conventional imaging method. In contrast, we observed no significant effect on the pattern of neurofibromas in the patients who did not follow MedDietCurcumin.

Epidemiological evidence and many case-control studies suggest that a Mediterranean diet plays a pivotal role in lowering the risk of several chronic diseases, including cardiovascular disease, neurodegenerative disease, diabetes, and cancer. A high intake of olive oil is considered a hallmark of the traditional Mediterranean diet. In this study, we used extra-virgin olive oil (EVO—the juice of the olive obtained solely by pressing and consumed without any further refining process) having >2000 mg/kg total phenol concentration. It has been reported that EVO consumption increases the monounsaturated fatty acid content in phospholipids and cholesterol esters by modifying the fatty acid composition of the plasma membrane, which influences the association of G proteins and PKCa with the lipid bilayer in elderly persons with type 2 diabetes [30]. The authors demonstrated that after consuming EVO for 4 weeks, the patients showed a significant increase in the total amount of monounsaturated fatty acid, mostly due to a rise in the proportion of oleic acid and a decrease in saturated fatty acids, which influences the membrane fluidity [31]. In addition, the EVO used contained a high level of naturally occurring phenolic compounds, the key feature of prevention of a number of diseases and pathological conditions (i.e., cancer and several aging-associated degenerative diseases) [32,33]. Increasing studies highlight the anti-proliferative and pro-apoptotic effects of the two major EVO components, oleuropein and hydroxytyrosol, on cancer cells and show that these effects stem from different mechanisms depending on the cell type. For example, these polyphenols are able to reduce angiogenesis via downregulation of cyclooxygenase-2 (COX-2) expression, prostanoid production and matrix metallopeptidase 9 (MMP-9) protein release, together with a reduction in intracellular ROS levels and NFκB activation [34,35,36,37]. Moreover, polyphenols stimulate apoptosis by activating pro-apoptotic Bcl-2 family members and PI3K/AKT signaling in pancreatic cancer and hepatoma cells, and increasing the c-Jun-N-terminal kinase (cJNK), p53, p21, Bax and cytochrome c cytoplasmic concentration in HeLa and cervix carcinoma cells [38,39,40]. Furthermore, in breast cancer cells, p53 or the G protein-coupled estrogen receptor 1/30 (GPER1/GPR30) pathway activation has also been shown, as well as the inhibition of the anti-apoptotic and pro-proliferation protein NFκB and cyclin D1, its main oncogenic target [41]. Moreover, the ability of curcumin to target multiple signaling pathways that are linked to tumorigenesis in NF1 represents a promising avenue for therapeutic intervention [42,43]. 

In particular, curcumin has been extensively studied for its role in Ras oncogenic signaling pathways, notably by abolishing the RAS-ERK signaling mechanism [44]. Depending on the cell type and stimulus, ERK activity mediates different antiproliferative events, such as apoptosis, autophagy and senescence in vitro and in vivo [45]. Findings in the last decade have demonstrated intriguing effects of curcumin in PI3K/AKT/mTOR signaling [46,47]. For instance, curcumin induces G2-M arrest and autophagy in malignant glioma cells through the inhibition of Akt/mTOR/p70S6K and activation of the extracellular signal-regulated kinase (ERK)1/2 pathways, suggesting that autophagy-mediated cell death might be pathway-specific [48]. In addition, preclinical studies show that curcumin induces apoptosis and G2–M arrest in cancer cells by generating superoxides, increasing caspase-3, caspase-7, and PARP cleavage, downregulating Akt phosphorylation, and upregulating p53 phosphorylation [49]. Curcumin also promotes selective tumor cell death and inhibits proliferation of a human hepatocellular carcinoma cell line (Huh7 cells), providing unequivocal evidence of intricate cross-talk between autophagy and cell death [50]. Another study demonstrated that curcumin increases the sensitivity of neurofibromin deficient MPNST cells to TRAIL (TNF-related apoptosis-inducing ligand), downregulating anti-apoptotic proteins [51,52]. Curcumin may also exert an anti-proliferative effect by decreasing the enzymatic activity of the epidermal growth factor receptor (EGF-R). In fact, NF1 Schwann cells exhibit an aberrant EGF-R expression, which has been linked to increased cellular proliferation and malignancy [53]. 

Unfortunately, while curcumin is one of the most used nutraceuticals, its utility as a therapeutic agent is limited by its poor water solubility, short biological half-life, and low bioavailability after oral administration in certain tissues. Therefore, various approaches including the use of adjuvants, liposomes, nanoparticles, phospholipid complexes and reformulation with various oils have been tried [54]. Interestingly, our six-month observation results show that only the patients who followed the MedDietCurcumin exhibited a plasmatic increase in curcumin concentration, indicating an improvement of curcuminoid bioavailability. We can speculate that the simultaneous presence of a high dietary concentration of EVO polyphenols and/or fatty acids contributes to this enhancement, and, consequently, to the positive effect on reducing NF1 symptoms. However, this study has some limitations, such as the small number of participants and the short-term exposure to MedDietCurcumin, but we can hypothesize that the regular consumption of polyphenol-rich olive oil and curcumin can maintain the effects observed in this study. 

## 5. Conclusions

NF1 presents a unique situation in which the existence of widespread neurofibromas, and their tendency to recur, calls for more effective and sustainable medical intervention than the simple surgical removal of individual lesions. The results presented in this study are of particular interest as they are the first clinical demonstration of the therapeutic activity on NF1 of curcumin in combination with a dietary approach rich in polyphenols.

As shown by our data, the plasma level of curcumin increased in patients following MedDietCurcumin; this suggests that curcuminoid bioavailability is positively influenced by polyphenol-rich foods in the Mediterranean diet. However, this synergism between curcumin and polyphenols needs to be confirmed on a larger cohort of NF1 patients.

Further studies are necessary to elucidate the molecular mechanisms by which MedDietCurcumin is able to reduce both the number and volume of neurofibromas. In addition, clinical trials involving new nanoformulations of curcumin conjugated with long-term observations may provide further data on the potential therapeutic role of curcumin in NF1 as well as in other inherited or sporadic cancer syndromes.

## Figures and Tables

**Table 1:** NF1 patient data.

Diet	Patient ID	Age	Sex	Familiar NF1	Sporadic NF1	Café-Au-Lait Spots Number > 6	Cutaneous or Subcutaneous Neurofibromas	Freckling	Lisch Nodules	Plexiform Neurofibroma	Scoliosis	Optic Pathway Glioma	Molecular Testing	Short Stature	Pathological Findings on Brain MRI
WesDiet	1 A.S.	38	M	yes	no	yes	yes	yes	yes	no	yes	yes	yes	no	T2-Weighted Hyperintensities (Ubo’s)
	2 A.F.	22	M	yes	no	yes	yes	yes	yes	yes	yes	yes	yes	no	T2-Weighted Hyperintensities (Ubo’s)
MedDiet	3 E.M.	27	M	yes	no	yes	yes	yes	yes	yes	no	no	yes	no	Fusiform aneurysms of both ICAs (ref)
	4 B.E.	18	M	no	yes	yes	yes	yes	yes	yes	yes	no	yes	yes	T2-Weighted Hyperintensities (Ubo’s)
	5 D.M.M.	22	M	no	yes	yes	yes	yes	yes	no	yes	no	yes	no	No signs
WesDietCurcumin	6 A.M.C.	41	F	yes	no	yes	yes	yes	yes	no	yes	no	no	no	T2-Weighted Hyperintensities (Ubo’s); vascular malformations (hypoplastic mid-distal portion of basilar artery and intra-cranial vertebral artery; intracavernous right carotid aneurysm)
	7 A.N.	59	M	no	yes	yes	yes	yes	yes	yes	yes	no	no	no	No signs
	8 A.F.	44	F	no	yes	yes	yes	yes	yes	no	no	no	no	no	T2-Weighted Hyperintensities (Ubo’s); hypoplasia of PCA
MedDietCurcumin	9 P.C.	54	M	no	yes	yes	yes	yes	yes	no	no	no	yes	no	No signs
	10 F.L.	27	F	no	yes	yes	yes	yes	yes	no	no	no	yes	no	No signs
	11 R.S.	50	M	no	yes	yes	yes	yes	yes	yes	yes	no	yes	no	Multiple foci of T2 hyper intensity (vascular gliosis)

Note: Ubo’s = unidentified bright objects; ICAs = internal carotid arteries; PCA = posterior cerebral artery; F: Female.

**Table 2:** Bioimpedentiometric and metabolic analysis in the course of follow-up.

Diet	Patient ID	Age (Years)	Sex	Time	% Cell Mass	% Tot Water	% Extracell Water	% Intracell Water	% Fat	% FFM ^1^	BMR ^1^	BMI ^1^	PA ^1^	NA/K
WesDiet	1 A.S.			*Baseline*	52.3	54	49.7	50.3	26.7	73.3	1235	25	5.7	1.2
		*38*	*M*	*6 months*	51.8	53	53.2	46.8	27.2	72.8	1198	25.4	5.5	1.3
	2 A.F.			*Baseline*	49.7	46.8	56.2	43.8	32.7	63.7	1065	28	6	1.2
		*22*	*M*	*6 months*	48.2	44.6	55.8	44.2	35.6	64.4	998.6	28.5	5.8	1.2
MedDiet	3 E.M.			*Baseline*	49.7	54	48.3	51.7	24.6	75.4	1324	26	5.5	1.2
		*27*	*M*	*6 months*	51.2	59.6	45.7	54.3	21.2	78.8	1435	23	6.1	0.9
	4 B.E.			*Baseline*	46.8	42.8	52	48	43.5	56.5	1129	36	4.8	1.3
		*18*	*M*	*6 months*	51.4	45.7	46	54	37.8	62.2	1326	30	5.8	1
	5 D.M.M.			*Baseline*	42.7	54.1	53.3	46.7	26.8	73.2	1223	27	5.9	1.1
		*22*	*M*	*6 months*	43.4	56.7	46.8	53.2	25	75	1276	26.7	6	1.1
WesDietCurcumin	6 A.M.C.			*Baseline*	50.9	58	48	52	21.4	79.6	1345.2	25	5.7	1.1
		*41*	*F*	*6 months*	51.6	59	47.2	52.8	20.9	79.1	1365	24.9	5.6	1.2
	7 A.N.			*Baseline*	47	48.8	54	46	38.6	61.4	1237	32	4.9	1.2
		*59*	*M*	*6 months*	47.1	47.6	55	45	38	62	1195	31.7	4.8	1.1
	8 A.F.			*Baseline*	49.2	48.7	50	50	34.4	65.6	1356	30	5.3	0.9
		*44*	*F*	*6 months*	50	48.2	48.5	51.5	33.6	66.4	1376.2	29.7	5.4	1
MedDietCurcumin	9 P.C.			*Baseline*	41.2	55.2	50.2	49.8	24.8	75.2	956.4	23	4.5	1.3
		*54*	*F*	*6 months*	51.5	59.3	48	52	19	81	1250.6	22	5.5	1.2
	10 F.L.			*Baseline*	49.1	45.4	49	51	38	62	1343.3	34	5.3	1
		*27*	*F*	*6 months*	57.1	48.3	45	55	34.1	65.9	1519.4	31	6.1	0.9
	11 R.S.			*Baseline*	50.2	59	47.2	52.8	19.4	80.6	1276.7	24	5.7	1.1
		*50*	*M*	*6 months*	52.2	64.4	47.6	52.4	12	88	1407.6	23	5.6	1.3

^1^ FFM: Fat-Free Mass; BMR: Basal Metabolic Rate; PA: Phase Angle; BMI: Body Mass Index.
